# Retraction: the cellular source for APOBEC3G's incorporation into HIV-1

**DOI:** 10.1186/1742-4690-8-88

**Published:** 2011-11-05

**Authors:** Jing Ma, Xiaoyu Li, Jian Xu, Quan Zhang, Zhenlong Liu, Pingping Jia, Jinming Zhou, Fei Guo, Xuefu You, Liyan Yu, Lixun Zhao, Jiandong Jiang, Shan Cen

**Affiliations:** 1 Institute of Medicinal Biotechnology, Chinese Academy of Medical Science, Beijing, PR China; 2 State Key Laboratory for Molecular Virology and Genetic Engineering, Institute of Pathogen Biology, Chinese Academy of Medical Science, Beijing, PR China; 3 Lady Davis Institute for Medical Research and McGill AIDS Centre, Jewish General Hospital, Montreal, Quebec, Canada; 4 Microbiology & Immunology, McGill University, Montreal, Quebec, Canada

## 

The authors would like to retract the article "The cellular source for APOBEC3G's incorporation into HIV-1"[1]. After publication of the article, the authors realized that the electrophoretic images in Figure 4D/E, showing a reproducible result of their previous publication [2] (cited as reference 6 [1]), was mistakenly taken from the same images used in [2]. In addition, Figure 2B is an unintended duplicate image of Figure 1B. This should have been avoided by the authors' careful proofreading of the manuscript. The authors apologize to the readers, reviewers, and editors of *Retrovirology *for publishing these erroneous data.

## References

[B1] Ma Jing Li Xiaoyu Xu Jian Zhang Quan Liu Zhenlong Jia Pingping Zhou Jinming Guo Fei You Xuefu Yu Liyan Zhao Lixun Jiang Jiandong Cen Shan The cellular source for APOBEC3G's incorporation into HIV-1 Retrovirology 2011 8 2 10.1186/1742-4690-8-2 21211018 PMC3024284

[B2] Cen S Guo F Niu M Saadatmand J Deflassieux J Kleiman L The interaction between HIV-1 Gag and APOBEC3G J Biol Chem 2004 279 33177 33184 10.1074/jbc.M402062200 15159405

